# Electroconvulsive Therapy in Psychiatric Disorders: A Narrative Review Exploring Neuroendocrine–Immune Therapeutic Mechanisms and Clinical Implications

**DOI:** 10.3390/ijms23136918

**Published:** 2022-06-22

**Authors:** Milagros Rojas, Daniela Ariza, Ángel Ortega, Manuel E. Riaño-Garzón, Mervin Chávez-Castillo, José Luis Pérez, Lorena Cudris-Torres, María Judith Bautista, Oscar Medina-Ortiz, Joselyn Rojas-Quintero, Valmore Bermúdez

**Affiliations:** 1 Endocrine and Metabolic Diseases Research Center, School of Medicine, University of Zulia, Maracaibo 4004, Venezuela; arizathings@gmail.com (D.A.); angelort94@gmail.com (Á.O.); mervinch12@gmail.com (M.C.-C.); joseluispv2811@gmail.com (J.L.P.); 2 Facultad de Ciencias Jurídicas y Sociales, Universidad Simón Bolívar, Cúcuta 540006, Colombia; m.riano@unisimonbolivar.edu.co (M.E.R.-G.); m.bautista@unisimonbolivar.edu.co (M.J.B.); 3 Psychiatric Hospital of Maracaibo, Maracaibo 4004, Venezuela; 4 Programa de Psicología, Fundación Universitaria del Área Andina, Valledupar 200001, Colombia; lcudris@areandina.edu.co; 5 Facultad de Medicina, Universidad de Santander, Cúcuta 540003, Colombia; o.medina@unisimonbolivar.edu.co; 6 Facultad de Ciencias de la Salud, Universidad Simón Bolívar, Barranquilla 080002, Colombia; 7 Division of Pulmonary and Critical Care Medicine, Brigham and Women’s Hospital, Harvard Medical School, Boston, MA 77054, USA; jrojasquintero@bwh.harvard.edu

**Keywords:** electroconvulsive therapy, mood disorders, neuroinflammatory diseases, neurogenesis, neurotransmitter agents, hippocampus, refractory depression, schizophrenia

## Abstract

Electroconvulsive therapy (ECT) is based on conducting an electrical current through the brain to stimulate it and trigger generalized convulsion activity with therapeutic ends. Due to the efficient use of ECT during the last years, interest in the molecular bases involved in its mechanism of action has increased. Therefore, different hypotheses have emerged. In this context, the goal of this review is to describe the neurobiological, endocrine, and immune mechanisms involved in ECT and to detail its clinical efficacy in different psychiatric pathologies. This is a narrative review in which an extensive literature search was performed on the Scopus, Embase, PubMed, ISI Web of Science, and Google Scholar databases from inception to February 2022. The terms “electroconvulsive therapy”, “neurobiological effects of electroconvulsive therapy”, “molecular mechanisms in electroconvulsive therapy”, and “psychiatric disorders” were among the keywords used in the search. The mechanisms of action of ECT include neurobiological function modifications and endocrine and immune changes that take place after ECT. Among these, the decrease in neural network hyperconnectivity, neuroinflammation reduction, neurogenesis promotion, modulation of different monoaminergic systems, and hypothalamus–hypophysis–adrenal and hypothalamus–hypophysis–thyroid axes normalization have been described. The majority of these elements are physiopathological components and therapeutic targets in different mental illnesses. Likewise, the use of ECT has recently expanded, with evidence of its use for other pathologies, such as Parkinson’s disease psychosis, malignant neuroleptic syndrome, post-traumatic stress disorder, and obsessive–compulsive disorder. In conclusion, there is sufficient evidence to support the efficacy of ECT in the treatment of different psychiatric disorders, potentially through immune, endocrine, and neurobiological systems.

## 1. Introduction

Electroconvulsive therapy (ECT) first appeared in 1934 when Hungarian neuropsychiatrist Ladislas Meduna used camphor in intramuscular oil to induce an epileptic episode in a patient who was suffering from catatonia [1]. In 1938, in Rome, Italy, Ugo Cerletti and Lucio Bini introduced electrical convulsions, decreasing the adverse effects of this therapy [2]. Thanks to these findings, modern ECT is based on conducting an electrical current through the brain to stimulate it and trigger a generalized convulsive activity with therapeutic effects [3].

The principles of this technique are the induction of a series of tonic–clonic seizures through an electric discharge in patients who have been previously examined and sedated with anesthetics and muscle relaxants. The relative contraindications and the number of sessions are determined by the specific pathology and the individual needs of each patient [4,5]. Likewise, there are different variants of this therapy, including modifications of the position of the electrodes and the amplitude of the electric pulse. In consequence, ECT can be classified as bilateral (bitemporal or bifrontal) and unilateral. Furthermore, it can be classified as brief or ultra-brief [6]. To date, multiple studies have demonstrated the efficacy of ECT in the treatment of different psychiatric and neurologic disorders [7]. Therefore, in 2018, the Spanish Society of Psychiatry established a consensus to determine in which cases this technique should be used [8]. Among the primary indications, major depressive disorder, manic episodes, schizophrenia, and catatonic states stand out. There are also possible secondary indications, such as obsessive–compulsive disorder (OCD) and post-traumatic stress disorder (PTSD), Parkinson’s disease (PD) psychosis, and malignant neuroleptic syndrome (MNS) [4,9,10,11,12].

However, despite its demonstrated efficacy and long-term effects, ECT continues to be considered one of the most debated and controversial treatments. A great part of the negative opinions emerged when it was first used in the medical setting, causing concern in the scientific community based on the multiple adverse effects observed. However, these effects have decreased considerably, thanks to the anesthetics and muscle relaxant inclusions [13]. Regardless, multiple negative opinions and stigma continue to exist, limiting its use [14]. This is mainly due to the erroneous understanding of ECT by the general population, where it is perceived as an antique or barbarian practice. Therefore, a great number of patients reject the therapy as they fear the stigma associated with it [15].

Despite this, there is robust clinical evidence that shows that ECT has a crucial role in the treatment of serious affective disorders, such as refractory major depressive disorder [16], refractory bipolar disorder [17,18], and refractory schizophrenia [19]. However, the molecular mechanisms of ECT are not particularly well known. Molecular psychiatry research has shown that its therapeutic effects take place through multiple neuroendocrine–immune mechanisms. These involve the deacceleration in the activities of neural networks, hyperconnectivity, neuroinflammation reduction, neurogenesis promotion, different monoaminergic systems modulation, and the normalization of the activity of the hypothalamus–pituitary–adrenal (HPA) and hypothalamus–pituitary–thyroid (HPT) axes. These are disruptions that are widely described in the pathophysiology of psychiatric disorders [20].

In this context, the goal of this review is to describe the neurobiological, endocrine, and immune mechanisms involved in ECT in mental illnesses, and to detail its clinical efficacy in different psychiatric pathologies.

## 2. Materials and Methods

This is a narrative review in which an extensive literature search was performed on the Scopus, Embase, PubMed, ISI Web of Science, and Google Scholar databases from inception to March 2022. The terms “electroconvulsive therapy”, “neurobiological effects of electroconvulsive therapy”, “electroconvulsive therapy and immune system”, “electroconvulsive therapy and the endocrine system”, “molecular mechanisms in electroconvulsive therapy”, and “electroconvulsive therapy and psychiatric disorders” were among the keywords used in the search. Afterward, the search was filtered using terms such as “humans” and “animals” as well as “clinical” and “preclinical”. Among the selection criteria for the studies, only those published within the past 35 years were included.

## 3. Results and Discussion

### 3.1. Molecular Mechanisms Involved in Electroconvulsive Therapy

Efficient ECT use during the last years has led to the emergence of different questions regarding the molecular basis involved in its action mechanism. Different hypotheses have been proposed, which are based on the neurologic function modifications and endocrine and immune changes that take place after ECT use in psychiatric disorders [21].

#### 3.1.1. Neurobiological Effects of Electroconvulsive Therapy

The ECT effects on the blood flow and metabolism of the cerebral cortex have been of great interest in current molecular psychiatry studies. Similarly, its impact on the neurogenesis and the neurotransmitter systems associated with the different mental illnesses’ physiopathology has been researched [20,22].

**Figure 1:**
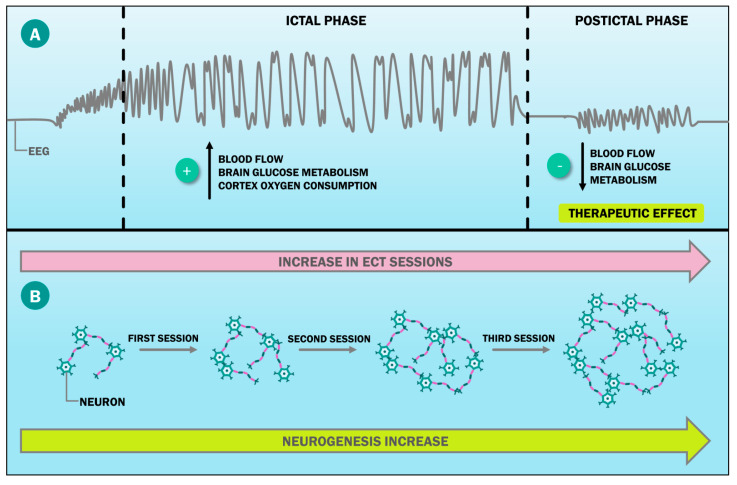
ECT neurobiological effects. ECT can cause therapeutic neurobiological effects, such as the following: (**A**) Changes in the metabolism of the brain cortex induced by different ECT phases. These are observable in the EEG, with the postictal phase being the one largely associated with the therapeutic ECT effect. (**B**) Induction of an increase in hippocampal neurogenesis, which is a dose-dependent effect. ECT: electroconvulsive therapy; EEG: electroencephalogram.

It has been proposed that part of the ECT neurobiological effects is based on its actions on the activity of the cerebral cortex, mainly in the frontal and temporal lobes [23]. The use of different imaging techniques has recently allowed for the identification of three stages seen during ECT [24]. The first one is the ictal period, in which there is a blood flow increase, glucose metabolism, and oxygen consumption in the cortex. The second one and the third one are the postictal period and the interictal period, respectively. The postictal period is the most important one as there is blood flow decrease as well as brain glucose metabolism, which is associated with ECT efficacy in the depression treatment (Figure 1A) [23,24,25].

This effect was demonstrated by Berggren et al., who evaluated the regional cerebral blood flow (rCBF) and depression scores of 49 patients before and after ECT and reported that 41 patients were classified as “with improvement” and 8 were classified as “no improvement.” This was based on a reduction of ≥50% in depression scores compared with pretreatment scores. Importantly, rCBF was significantly higher in the left temporal lobe in patients who did not improve after ECT and who also presented with more sustained depression and anxiety features [25].

In this sense, depression has also been associated with hyperconnectivity of the brain network and the delta and alpha waves present in the electroencephalogram (EEG). Particularly, hyperconnectivity between the anterior temporal cortex and the subgenual cortex has been observed. Studies such as the one performed by Deng et al., have shown that ECT would be able to produce low-frequency wave increase and hyperconnectivity decrease among the brain networks described in the depression cases [26,27]. Similarly, Leave et al., showed that connectivity between the lobes before ECT could predict its antidepressant effect. They evaluated basal brain activity, connectivity, and depression symptoms before and after ECT use in 46 patients, supporting the importance of frontotemporal effects to achieve clinical changes [28].

It has also been reported that ECT acts on the neuronal structure volume. According to Joshi et al., part of its therapeutic action is achieved, thanks to its capacity to promote neurogenesis in the hippocampus and the amygdala. This group evaluated these structures in 32 control patients and 43 patients with major depression before ECT after the first session and after a week of completing the ECT treatment series. They observed that before the beginning of the treatment, the patients with major depression had smaller hippocampal volumes than the control patients. After ECT, the hippocampus and the amygdala sizes increased, and this was associated with symptom improvement. Similarly, the hippocampus volume before ECT predicted the clinical response to it. In consequence, it has been hypothesized that patients with a lower hippocampus volume before ECT tend to present with a higher volume increase and a better clinical response [29].

Likewise, different authors, such as Oltedal et al., and Cao et al., have proposed that ECT acts by increasing neurogenesis in subfields such as the dentate gyrus at the granular and molecular cell layers, the subiculum, and the cornus ammonis. This increase is dose dependent, meaning that the greater the ECT dose, the larger the volume increase (Figure 1B) [30,31]. Therefore, the current understanding is that there is a causal relationship between the neurogenesis decrease in these areas and psychiatric diseases such as major depression, schizophrenia, and bipolar disorder [29,32,33].

However, in recent studies, it has been observed that the hippocampal volume increase is not a perfect biomarker to determine the ECT clinical results. Such is the case in Cao et al., who reported that patients with a greater increase in hippocampal volume presented with a poorer response to the treatment. This was seen more frequently in patients with few positive clinical results in which a greater number of the ECT sessions were used. Similarly, it was found that lower volumes in the hippocampus-specific subfields, such as the cornu ammonis 3 and 4, the granular layer, the molecular layer, and the subiculum, are associated with a better response to ECT [31]. In a meta-analysis performed by Wilkinson et al., in which nine studies were included for a total of 174 patients, researchers proposed that the neurogenesis and volume increase could be an epiphenomenon in ECT and would not entirely represent the entirety of its neurobiological mechanism of action [30,34].

Therefore, during the last decade, the hypothesis that part of the therapeutic effects of ECT could be caused by a metabolism modification and the function normalization of multiple neurotransmitters has emerged. This would include noradrenaline (NA), glutamate, γ-aminobutyric acid (GABA), dopamine (DA), serotonin, and brain-derived neurotrophic factor (BDNF) [22].

**Figure 2:**
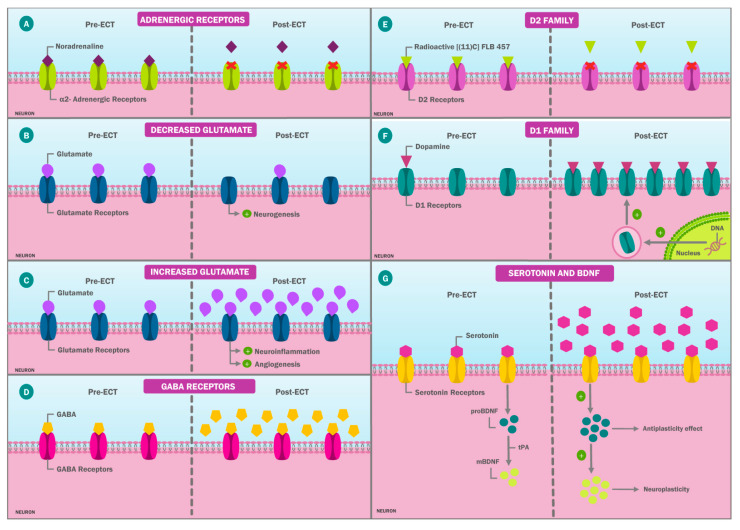
ECT effects on neurotransmitters. ECT has a variety of effects on important neurotransmitters and/or their receptors. These include (**A**) the ECT noradrenergic effect that decreases the α2 adrenergic receptors’ affinity to their ligands, noradrenaline. Likewise, ECT has glutaminergic effects that could be generated through some of the following pathways: (**B**) a decreases in the glutamate concentration after ECT with a consequent increase in neurogenesis or (**C**) an increase in the glutamate concentration after ECT, which is associated not only with the neuroinflammation but also with the promotion of angiogenesis, which is part of neurogenesis. Regardless, (**D**) another effect of ECT involves GABA because the levels of this neurotransmitter increase after ECT. Furthermore, ECT generates changes in the dopaminergic receptor family, such as (**E**) a decrease in the affinity of the D2 receptor to its ligand [(11)C]FLB 457 radioactive, and (**F**) ECT increases protein synthesis and the gene expression codifying for the D1 receptor. It also improves the D1 receptors’ affinity to their ligands. Finally, (**G**) this therapy has effects on the serotoninergic systems and BDNF through an increase in the serotonin levels, which promotes neuroplasticity through the BDNF synthesis stimulation. However, ECT also causes an increase in the proBDNF levels, which promotes neuronal apoptosis and has antiplasticity effects, which makes the tPA role a crucial one as it is an important element in the transformation from proBDNF to mBDNF. The latter is the one responsible for the neuroplasticity promotion. ECT: electroconvulsive therapy; BDNF: brain-derived neurotrophic factor; proBDNF: precursor isoform of BDNF; tPA: tissue plasminogen activator.

In this context, it has been proposed that the noradrenergic activity modification in major depression is caused by alterations in the adrenergic receptors, especially in the α2-adrenergic receptors [35,36]. It has been hypothesized that an affinity increase could be part of the disease pathophysiology [37]. Based on this, Lillethorup et al., studied the adrenergic α2 receptors’ affinity in mice with a depression animal model. The said affinity was measured after the ECT application, and it was compared with the affinity of the same receptors in a control group. It was found that after the ECT application, adrenergic α2 cortical receptors showed a decreased affinity, suggesting that this could be a mechanism through which ECT achieves its therapeutic effects (Figure 2A) [38].

Alternatively, it has been proposed that glutamatergic system dysfunction could be involved in the neuropsychiatric disease physiopathology, including affective disorders, schizophrenia, and even Alzheimer’s disease (AD) [39,40]. Studies based on this hypothesis are controversial. On the one hand, some researchers, such as Niau et al., have reported a glutamate concentration decrease after ECT, which has been associated with a neurogenesis increase and a consequent clinical improvement in neuropsychiatric diseases in which neurogenesis is impaired, such as AD (Figure 2B) [41]. On the other hand, Cano et al., reported a glutamate concentration increase after ECT, an increase that the authors associated with neuroinflammation, which is an adverse effect of the therapy. In addition, it is proposed that glutamate increase could promote angiogenesis, which is considered part of the neurogenesis process (Figure 2C) [42].

In consequence, the effect of ECT on the glutamatergic system is unclear; however, Abbott et al., reviewed 26 studies performed between 2002 and 2013, observing the consistency in the results supporting the ECT effect on the glutamatergic system through a concentration increase or normalization. This was also associated with the clinical improvement, finding increased glutamate levels only in patients who responded to the treatment. Therefore, it is presumed that glutamate and the remaining neurotransmitters play an important role in the ECT action mechanism [21,43].

In the case of GABA, it has been proposed that ECT can increase its concentration, leading to a potentiation of its effects. To examine this hypothesis, Xia et al., performed a study in which there was a control group, a group of patients with schizophrenia who received only antipsychotic treatment, and a group formed by patients with schizophrenia who received ECT. The GABA concentration was determined for all groups, and these levels were also measured in groups 2 and 3 after receiving the treatment. They found that GABA levels were lower in schizophrenic patients compared with nonschizophrenic patients. They also reported that after a 4-week treatment with ECT, the GABA levels increased in this group, which did not happen in the patients who only received treatment with antipsychotic drugs (Figure 2D) [44].

Knudsen et al., performed similar research on patients diagnosed with depression or bipolar disorder; however, they did not report significant differences between the GABA levels in patients with the diagnosis compared with healthy patients. Similarly, no difference was observed in the GABA concentrations after ECT. This highlights the difficulty of determining the ECT effect on the GABAergic system due to the inconsistencies observed in the study results [45]. In their review of 26 studies performed between 2002 and 2013, Abbott et al., found that their results coincided, showing an increase in GABA levels in the occipital cortex, in the blood, or in the cerebrospinal fluid after ECT. However, these studies do not coincide in the correlation between these changes and clinical response, supporting the ECT effect on the GABAergic system without determining whether this effect mediates its therapeutic action [43].

The dopaminergic system has also been studied due to the possible role of DA in the different neuropsychiatric diseases’ physiopathology, such as depression and schizophrenia, as well as its protagonist role in PD. These are all diseases in which ECT leads to an evident clinical improvement, leading to a theory of ECT having a regulating effect on DA activity [46,47,48,49].

However, it has not been established at which level of the DA pathway this effect would take place. Recently, the ECT impact on dopaminergic receptors has been studied, including the D1 and D2 receptor families. Saijo et al., determined the ECT effect on D2 receptors in patients diagnosed with depression. Their findings showed that after a series of six to seven ECT treatments, the union of these receptors to radioactive [(11)C]FLB 457 decreased. They proposed that these receptors could therefore be part of the ECT action mechanism; however, these findings have not been constant among studies, which is why this theory has not been confirmed (Figure 2E) [50,51,52].

On the other hand, Kobayashi et al., reported that ECT leads to a rapid and long-lasting dopaminergic modulation improvement, especially in the hippocampus. This modulation is attributed to protein synthesis changes and the expression of the genes that code for dopaminergic receptors. They also found that ECT causes positive regulation in the genes for the D1 receptor family, including D1 (Drd1) and D5 (Drd5) receptors. This change was not seen in the genes coding for D2 receptors [53]. Similarly, they described that these rapid and long-lasting changes are more frequently present in the mossy fibers’ synapses of the hippocampus within the CA3, which is an area that shows greater sensitivity to ECT neuronal stimulation compared with other areas of the brain (Figure 2F) [53].

Likewise, Landau et al., found that ECT could improve the affinity of D1 receptors to their ligands. This improvement would last for up to 6 weeks, which coincides with the temporary ECT effect on depression and PD. These affinity changes were described as an acute ECT effect, which was observed 24–48 h after the treatment. However, the authors did not discard that this phenomenon is caused by a DA release increase. In this same study, it was described that the more prolonged ECT effects could be caused by an increased D1 receptor number, which can result from the rise in the dendritic column number and/or size. This suggests a neuroplastic effect that is instrumental to obtain these results [54].

It has also been proposed that ECT could have an impact on the serotoninergic system modulation, which would lead to neuroplasticity changes [55,56,57]. According to this theory, ECT would lead to a serotonin increase and a reuptake decrease together with a rise in the expression and serotonin receptors’ affinity, potentiating the neurotransmitter functions [22,58,59].

In this sense, Kronenberg et al., performed a study in which they induced electric convulsions (ECs) in two groups of mice, a wild group and a group deficient in tryptophan hydroxylase 2 presenting with serotonin depletion in 2018. The second group showed symptoms very similar to the ones typically seen in patients with depression. Both groups received five ECs a day for 3 days, and the EC effect on adult neurogenesis and the brain-derived neurotrophic factor (BDNF) was evaluated. It was reported that both neurogenesis and BDNF were lower in mice (Tph2^−/−^) compared with wild mice. The authors concluded that serotoninergic signaling is a requirement that mediates key ECT neurobiological effects [60].

However, the serotonin role in neuroplasticity is controversial, and it has been proposed that the neurotransmitter decrease or total absence could favor or, at least, have no adverse effect on neurogenesis and neuronal survival. This was also reported by Kronenberg et al., in 2016, who measured BDNF concentration in the hippocampus and the cortex of the two mice models. The first (Tph2^−/−^) model had no brain serotonin. The second model with serotonin transporter (SERT^−/−^) deficiency had no neurotransmitter reuptake, leading to an increase in serotonin concentration. It was observed that BDNF concentration increased in the hippocampus and the brain cortex of the (Tph2^−/−^) mice but not in the (SERT^−/−^) mice. It was concluded that the brain serotonin absence induces BDNF expression; therefore, the ECT effect on the serotoninergic system and neurogenesis is refutable and requires further research in the future [61].

Finally, it has been proposed that a decrease in BDNF concentrations plays an important role in the different pathophysiology of different neuropsychiatric disorders [62,63,64]. Rocha et al., and Li et al., reported that ECT leads to a BDNF concentration increase in both the central and the peripheral nervous system, thus increasing the precursor isoform (proBDNF) concentration and the mature BDNF (mBDNF) by increasing the tissue plasminogen activator (tPA) levels. This is a key element in the proBDNF transformation into mBDNF [65,66]. Although this phenomenon could explain the therapeutic ECT effect, it would also increase the proBDNF concentration, which could lead to negative cognitive effects due to proBDNF coupling preferentially to the neurotrophin receptor p75. This leads to neuronal apoptosis and antiplasticity effects, especially in the CA1 area of the hippocampus, explaining the negative cognitive ECT effects (Figure 2G) [67,68].

However, not all studies have been in favor of this hypothesis. Ryan et al., evaluated the BDNF serum concentrations in 50 control patients and 61 patients with depression. No differences between the two groups were found. Likewise, they measured serum BDNF levels in patients with depression before and after ECT, reporting that there was no significant difference after ECT. In addition, Sorri et al., reported a decrease in BDNF serum concentration during ECT. Therefore, authors such as van Zuthpen have called into question the role of BDNF serum levels as a biomarker to determine the therapeutic ECT effect, as the specificity and sensitivity of this marker are deemed too low [69,70,71,72].

A meta-analysis by Polyakova et al., was composed of studies in which BDNF concentrations at the central and peripheric levels were determined before and after ECT. The studies included were published before 2014. It was reported that ECT increased the genetic expression and synthesis of BDNF, which is positively associated with the treatment number. This corresponds to a second meta-analysis, performed by Rocha et al., which was composed of 261 studies performed between 1990 and 2016. It was reported that BDNF concentrations increased after ECT, recommending the use of BDNF as a potential biomarker to assess treatment response [65,73].

#### 3.1.2. Impact of ECT on the Endocrine System

A considerable portion of the different psychiatric diseases’ pathophysiologic model stems from the neural diathesis–stress model, in which the HPA axis hyperactivity maintains high cortisol levels [74,75]. This causes multiple neurologic alterations present in psychiatric diseases, such as a decrease in the astrocyte marker levels, BDNF glial fibrillary acidic protein (GFAP), brain volume modifications, and even cognitive alterations [74,76].

In addition to this, multiple studies have reported clinical changes in the HPA axis after the ECT use [74,77,78], improving the pathologies’ symptoms, such as depression [79,80], schizophrenia [81], and bipolar disorder [82,83]. Burgese et al., administered 12 ECT sessions to patients with depression while measuring their serum cortisol levels before starting ECT, after the seventh session, and after the last session. Furthermore, they compared these levels with control patients. They reported that ECT led to a decrease in cortisol levels in patients with depression, reaching the same concentrations as the control group and showing a clinical improvement [84].

Similarly, according to a study performed by O’Donovan et al., ECT can increase BDNF and GFAP expression in the cortisol model of depression. Likewise, Mickey et al., described that the cortisol trajectory in patients’ hair with refractory depression can predict their responses to ECT, finding that patients with an ascendant trajectory have a better response than those with a descendant trajectory [76,85].

Conversely, even though these results have been reported by different studies, other studies have contradicted these findings. Such is the case of Kyeremanteng et al., who applied ECT to mice with an animal model of depression for 5 days. They found that there was a corticotropin-releasing factor (CRF) increase in the hippocampus and outside of it, in the frontal cortex, and in the neocortex. In addition to this, the mice presented with retrograde amnesia, which is a frequent adverse ECT effect. A temporal association between CRF blood levels and the antidepressant effect and retrograde amnesia induced by ECT was also observed. The authors suggest that these results are a consequence of the possible anxiolytic CRF effect and its capacity to antagonize CRF1 receptors, which have been associated with memory loss after prolonged stress exposure [86].

As a result of this research and conclusions, the ECT effect on the HPA has not been firmly determined. However, the majority of studies appear to support the hypothesis that ECT decreases HPA hyperactivity. In addition, the evidence gives a starring role to HPA in the ECT action mechanism [21].

Other hormones and axes have been involved in the ECT action mechanism. The HPT axis stands out in this context. Abnormalities described in thyroid-stimulating hormone (TSH) levels have been associated with an increase in vulnerability to the depression symptoms’ onset and AD development. In this context, Dikes et al., reported an acute increase in serum TSH level increase after ECT. However, these changes have not been associated with the clinical improvement of patients. Alternatively, Decima et al., reported that after ECT, there was a decrease in the TSH response to the thyrotropin-releasing hormone (TRH). In addition, Esel et al., observed that even though ECT could acutely increase TSH levels, the hormone serum levels of this hormone decreased 60 min after a session [87,88,89,90,91,92,93,94,95,96].

Other elements of this axis have also been studied, particularly the thyroid hormones thyroxine (T4) and triiodothyronine (T3). It has been hypothesized that ECT could decrease the T4 free fraction, which would have a therapeutic effect considering that increased levels of this hormone have been reported in patients with schizophrenia and with a suicidal ideation presence. Despite this, the ECT effect on the HPT axis continues to be controversial [88,91,97].

#### 3.1.3. Effects of ECT on the Immune System

**Figure 3:**
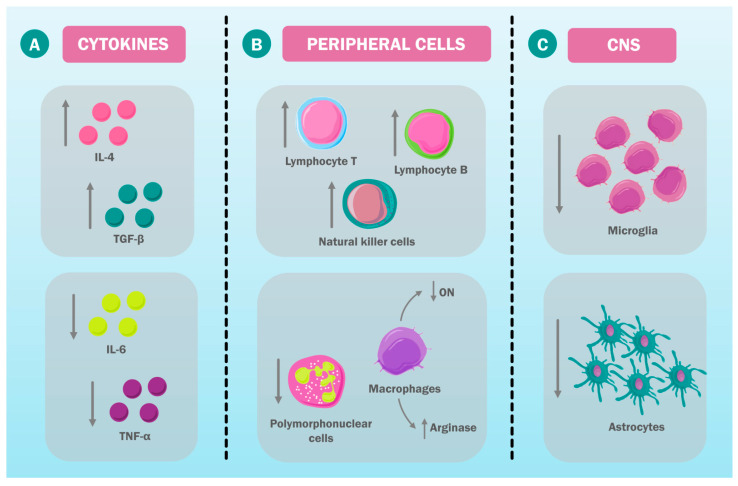
ECT immunologic effects. ECT has an impact on different elements of the immune system, including the following: (**A**) the proinflammatory and anti-inflammatory cytokine levels, (**B**) the peripheral immune system cells’ proliferation and activity, and (**C**) the immune CNS systems’ activity, such as microglia and astrocytes. IL-6: interleukin-6; TNF-α: tumor necrosis factor alpha; IL-4: interleukin 4; TGF-β: transforming growth factor beta; NO: nitric oxide.

The inflammatory hypothesis is one of the most recent pathophysiological mechanisms that have been proposed in the context of neuropsychiatric disorders. More specifically, inflammation has been associated with changes in the HPA axis and neurogenesis. Therefore, the immunologic ECT impact, specifically its effects on cytokines as well as on microglia activity, has gained relevance in recent years (Figure 3) [98,99].

Cytokine concentration alterations have been identified in patients with major depressive disorder (MDD), bipolar disorder, and schizophrenia. The key cytokines in this context are interleukin-6 (IL-6) and tumor necrosis factor α (TNF-α), which could be involved in the ECT action mechanism. Regarding IL-6, Zincir et al., measured its levels in 50 patients with depression before and after ECT and compared them with 30 control patients. They reported that IL-6 levels were higher in patients with depression compared with control patients before ECT. After the therapy, the difference decreased [100].

In addition, Järventausta et al., reported that ECT causes an acute IL-6 level increase. However, these levels decreased days after the treatment. After a full ECT series, IL-6 levels were significantly decreased in patients that showed clinical improvement, but not in patients who did not show clinical improvement. This seems to indicate that the long-term ECT effect on IL-6 is correlated with its clinical results. Therefore, IL-6 could have an important role in the ECT action mechanism, and it would also serve as a biomarker to determine its effects [101,102].

Likewise, TNF-α has been associated with the pathogenesis of mood disorders and the mechanism of action of ECT. It has been proposed that this therapy could have an anti-inflammatory effect, temporarily decreasing TNF-α levels. Similarly, it has been proposed that the acute TNF-α level’s decrease would be associated with the symptomatic improvement of patients with depression [103]. The cytokines’ role has not only been described in the ECT context and depression. In the schizophrenia context, Kartalci et al., researched the serum levels of interleukin-4 (IL-4), transforming growth factor β (TGF-β), myeloperoxidase (MPO), and nuclear factor-κB (NF-κB). They reported that NF-κB levels were higher in patients with schizophrenia, while TGF-β levels were lower than those observed in control patients. No significant changes were reported in MPO and IL-4 levels. Similarly, they studied the serum levels of these cytokines in patients after the ECT administration, and they found that the clinical improvement seen during the treatment was accompanied by a gradual IL-4 and TGF-β increase. However, no changes in MPO and NF-κB levels were found. In consequence, an anti-inflammatory role for ECT through its action on cytokines has been proposed [104].

This activity could also be mediated by the immune system’s peripheral cells. Chaturvedi et al., reported an increase in the leucocytes’ total number and the lymphocytes’ percentage as well as a decrease in the percentage of the polymorphonuclear cells after ECT administration. Similarly, Kronfol et al., measured the levels of natural killer cells in the peripheral blood. It was found that ECT caused an increase of these lymphocytes only minutes after the session, reaching its peak 10–30 min later and starting to decrease after 60 min [105,106].

A summary of the ECT effects that have been observed in different psychiatric illnesses is presented in Table 1.

Macrophages have a role in the ECT impact on the immune system regulation. Roman et al., reported the changes in the biological properties of peritoneal macrophages in mice that received ECT once a day for 10 days. The peritoneal macrophages’ examination revealed a decrease in their proinflammatory properties and an increase in their arginase activity and a decrease in the synthesis of nitric oxide (NO). Furthermore, the same authors determined in another study that a NO synthesis decrease causes an increase in the T and B lymphocytes’ proliferative response. Additionally, they highlighted that these changes took place after a series of electric convulsion applications; therefore, time is required for these changes to appear [107,108].

Peripheral macrophages would not be the only ones involved in the immune ECT effects. Macrophages in the CNS would also be related to the ECT action mechanism as physiologic changes in these cells have been observed after ECT administration [109]. It has been observed that macrophage recruitment in the hippocampal circulation takes place as a consequence of ECT. However, no signs of inflammation in the neuronal parenchyma have been reported. On the contrary, it has been proposed that ECT has an immunosuppressant effect on immune cells at the central level, modulating the innate immunologic system. This is relevant due to the hypothesis that neuropsychiatric diseases, such as depression and schizophrenia, are caused by microglia and astrocyte hyperactivity, which could decrease neurogenesis and neuroplasticity [110].

For this reason, the inhibiting ECT effect on these cells would be beneficial. This has been observed in animal models of depression and schizophrenia. Limo et al., confirmed these changes through the microglial gene CD11b and astrocyte-expressed GFAP measurement. Both of these are distinctive activity markers for these cells. This study found that the CD11b levels in the dentate gyrus and the CA1 and CA3 areas were higher in mice with an animal model of schizophrenia compared with control mice. Similarly, GFAP levels in the GD and the CA1 area were higher in mice models than in the control group, indicating that microglial and astrocyte activity was higher in schizophrenic mice. However, these levels decreased after ECT sessions, indicating a microglial and astrocyte activity inhibition. In a study performed by Kranaster et al., this inhibition as an ECT consequence was also reported in humans, reinforcing the hypothesis that ECT exerts its therapeutic effect through immunomodulation [110,111,112,113,114].

### 3.2. Unifying Neuroendocrine–Immune Hypothesis of ECT in Psychiatric Disorders

Different hypotheses regarding the mechanisms of action of ECT have been explored throughout the years to explain how one technique can have a therapeutic effect in such a wide variety of disorders despite their notable clinical and pathophysiological differences. These hypotheses are based on findings obtained through preclinical and clinical research that, far from reporting homogenous results, show the effects of ECT on the CNS, which go from structural changes to functional modifications and even changes in other systems, such as the endocrine and immune systems [21].

For example, as an explanation for these phenomena, Wilkinson et al., proposed that the increase in volume in neuronal structures observed after the use of ECT could be an epiphenomenon that accompanies the true mechanisms involved in the clinical improvements shown by patients, without direct effect on clinical improvement [34]. Conversely, Joshi et al., reported that in patients with depression, the increase in the volume of the hypothalamus and the amygdala after ECT would be directly related to clinical improvement. Furthermore, it was suggested that the promotion of neurogenesis in these structures at the same time as a decrease in the activity of the frontal and temporal cortexes results in clinical improvement, as was demonstrated by Beggren et al., and Leave et al., Therefore, ECT would be countering two important changes in the pathophysiology of depression, referring to the decrease in the size of neuronal structures and frontotemporal hyperconnectivity and hyperactivity [25,28,29].

On the other hand, Lillethorup et al., and Abbott et al., proposed that ECT decreases neuronal activity through the reduction of the affinity of α2 adrenergic receptors to noradrenaline and the increase in GABA levels, respectively. Therefore, the hypothesis of interconnectivity could coexist or even be related to the monoamine hypothesis [38,43]. Likewise, the latter would be associated with neurogenesis, as Njau et al., Cano et al., and Abbott et al., found that ECT has an impact on the glutaminergic system by regulating glutamate levels. Therefore, there is an improvement in the symptoms of patients with depression, schizophrenia, or Alzheimer’s disease and promotion of angiogenesis and neurogenesis [41,42,43].

Other monoamines that could contribute to the neuroplasticity and neurogenesis induced by ECT are dopamine and serotonin. This was the reasoning for the studies of ECT on the dopaminergic system performed by Kobayashi et al., and Landau et al., reporting that this treatment leads to an increase in the expression and affinity of D1 receptors. Meanwhile, Kronenberg et al., demonstrated that the serotoninergic system plays a primary role in the mechanism of action of ECT [53,54,60].

However, the monoamine system is not the only one considered in the context of the mechanisms of action of ECT-induced neurogenesis. Burgese et al., proposed that a dysregulation in the HPA axis would lead to a decrease in cerebral volume. Therefore, by regulating the cortisol levels through ECT, this effect would be countered [84]. In addition, all the previously mentioned mechanisms could be grouped within the immunologic effects of ECT, as inflammation is associated with HPA and neurogenesis disruptions that could be countered by ECT. This would be achieved through a decrease in the levels of IL-6 and TNFα and the levels of polymorphonuclear cells as well as the increase in IL-4, TGF-β, and total leucocyte and lymphocyte percentages, according to studies performed by Zincir et al., Järventausta et al., Kartalci et al., and Chaturvedi et al. [100,102,104,105].

As a result, even though these mechanisms were proposed and researched individually, there is a current understanding regarding the close relationship that exists among these different systems. Therefore, the possibility of a new perspective in the study of the mechanisms of ECT appears, in which the evidence for one hypothesis does not necessarily contradict a second hypothesis. Instead of determining which hypothesis has the most evidence in its favor, it is proposed that ECT can act in unison through different mechanisms that are related to each other, which leads to a wide effect on the aforementioned systems, explaining the vast effect of ECT across multiple disorders [115].

That said, numerous details regarding the effect of ECT on each of the neuropsychiatric diseases in which it could be used are still unknown. More specifically, there is still the question of which mechanism corresponds to the effects observed in each disease. Furthermore, it has not been established whether these mechanisms can vary when the technique is modified according to the position of the electrodes, the magnitude and duration of the electric wave, the use of anesthetics and muscle relaxants, the frequency of the sessions, and the use of coadjuvant drugs. The majority of these studies in which these variations are accounted for focus on clinical response and not on the neurobiological pathways [116,117]. An example of this is that greater efficacy in patients with depression has been observed when bilateral ECT is used over unilateral ECT. However, the mechanisms for these observations have not been explained. Therefore, the need for additional research on variations like these is evident [118].

Finally, important gaps in knowledge are present in clinical practice, together with a considerable stigma that surrounds ECT. This limits its use and study, which leads to the lack of precise guidelines on the use of ECT in the management of neuropsychiatric diseases, with a lack of consensus regarding when to use ECT in the treatment algorithm. ECT continues to be a subject of study in modern medicine, and the understanding of its uses in other neuropsychiatric diseases is expected to grow in the coming years [119,120,121].

### 3.3. From Molecular Mechanisms to Clinical Evidence: Impact of ECT on Psychiatric Diseases

The majority of the neuroendocrine–immune mechanisms of ECT are physiopathological components and therapeutic targets in different mental illnesses. Therefore, its clinical impact on psychiatric diseases in which these mechanisms could lead to therapeutic effects is relevant.

During the last decades, the use of ECT has had a crucial role in the treatment of severe affective disorders, such as refractory major depression, refractory bipolar disorder, and refractory schizophrenia. More recently, clinical evidence has also been reported in other serious psychiatric pathologies with diverse physiopathological mechanisms, such as PD psychosis, MNS, OCD, and PTSD.

#### 3.3.1. Refractory Major Depressive Disorder

ECT is currently considered the most effective treatment for refractory MDD [122]. In 1985, the first meta-analysis evaluating the efficacy of ECT was performed, reporting that this was a treatment superior to antidepressant medication and simulated ECT or placebo [123]. The number of clinical assays evaluating the use of ECT has exponentially increased since then, and ECT itself has considerably advanced as a procedure [118,124]. Three decades later, these initial findings have been confirmed by the meta-analysis performed by Han Kho et al. [125]. However, there is no clear consensus on the position of ECT in the depression treatment protocol. While certain authors recommended that depression with psychotic characteristics can be treated with antidepressants in monotherapy or in combination with antipsychotics [126,127], other authors proposed that the first-line treatment should be ECT [128,129]. In clinical practice, ECT is commonly used to treat patients with refractory depression, with a response rate of 70% in patients with depression and 58% in patients with depression resistant to treatment [130]. Greater efficacy has been described in bilateral ECT compared with unilateral ECT [118,124].

Acute results with ECT have been very effective in inducing depression remission [16,131]. However, the recurrence rate is alarmingly high once the treatment is finished, even after follow-up treatment with medication is administrated [132,133,134]. Therefore, ECT continuation (ECT-c) and ECT maintenance (ECT-m) use have been recently studied, showing efficacy in depression relapse and recurrence prevention [135]. ECT-c refers to continued treatment during the first 6 months after remission, and ECT-m refers to maintenance beyond 6 months [136]. Elias et al. [135] performed a meta-analysis that included 5 studies and 436 patients, reporting that ECT-c and ECT-m, together with medication in periods of 6 months and 1 year, were significantly associated with fewer relapses and recurrences compared with those that only received pharmacological treatment.

The following section summarizes the key clinical evidence of the impact of ECT as a mental disorder treatment (Table 2).

#### 3.3.2. Schizophrenia

The first patients treated with ECT were patients with schizophrenia [138]. This therapy was the most frequently used one for acute psychosis until 1952, when it was entirely displaced by pharmacological treatment [139]. Regardless, its use has recently gained considerable interest among physicians due to its usefulness in refractory schizophrenia [140]. The criteria for ECT prescription are not consistent in the current literature; however, different treatment guidelines propose its indication in patients with schizophrenia with catatonia symptoms, as additional support for pharmacotherapy when there is resistance to treatment, and in patients with a prior positive response to ECT [136,141,142]. It has been reported that treatment resistance is the most common condition in which ECT is prescribed [143]. Furthermore, the symptomatic and cognitive response has been higher when bifrontal electrodes are used [144]. A recent meta-analysis performed by Ahmed et al. [19] included 23 studies and 1179 patients, reporting significant improvement in patients with schizophrenia resistant to pharmacological treatment who received ECT + clozapine (SMD: −1.504; CI: 95%) and ECT + nonclozapine antipsychotics (SMD: −0.891; CI: 95%).

In addition, numerous studies have reported the beneficial ECT impact on catatonic schizophrenia. Some authors have even reported that it has a faster response rate than in noncatatonic schizophrenia [145]. Suzuki et al., evaluated the short-term and long-term ECT use in catatonic schizophrenia resistant to treatment, reporting that 100% of patients were responsive to this treatment. However, relapses took place in 7 of the patients during the first 6 months of treatment. The recurrence rate 1 year later was 63.6%, despite continued pharmacotherapy [146]. These patients received a second therapy cycle, followed by ECT-m for a year. Four of them continued to be in remission [147]. Those who relapsed once again were successfully treated by adjusting the frequency of the sessions [148].

Recent research studies have explored ECT efficacy in teenagers and young adults with schizophrenic disorders refractory to pharmacotherapy. In a prospective study, Suzuki et al. [149] examined the acute ECT short-term effects and its safety in young adults in their first schizophrenic episode that was not medically treatable. A significant decrease in clinical symptoms was reported 1 week after the final ECT session. Likewise, De la Serna et al. [150] studied the long-term ECT cognitive effects in a sample of teenage patients with schizophrenia, in which no significant differences were observed between the ECT group and the non-ECT group. Furthermore, another study reported a notable decrease in the hospitalization length of patients treated with ECT + antipsychotics compared with those treated only with antipsychotics. Significant improvements were seen in positive symptoms and in general psychopathology [151].

#### 3.3.3. Bipolar Disorder

The value of ECT in the treatment of BD has a variable amount of clinical evidence in the context of its efficacy in mania, bipolar depression, and mixed states. Different treatment guidelines for BD propose the use of ECT as a first-line treatment only in very serious cases or patients resistant to pharmacotherapy [152,153,154,155,156]. Other guidelines indicate ECT as a second-line or third-line treatment in pregnant patients with catatonia, psychotic characteristics, or bipolar depression [157].

Although ECT has been more widely used in the treatment of unipolar depression, its use in bipolar depression has expanded in recent years [137]. The main obstacle in this context has been concerns regarding a possible change to a manic or hypomanic state. However, it has been reported that clinical improvement is more effectively achieved with ECT than with pharmacotherapy in bipolar depression [137,158,159]. In a meta-analysis that included 19 studies, Bahji et al. [137] reported a response rate of 77.1% and a remission rate of 52.3% in patients with bipolar depression treated with ECT. Furthermore, response rates and the speed of the response were higher in individuals with bipolar depression compared with those with unipolar depression. Recently, a multicentric, randomized, controlled assay performed by Schoeyen et al., compared the efficacy of ECT with that of pharmacological treatment in 73 patients with resistant bipolar depression. It was reported that the acute bipolar depression phase can have a better response to ECT than to pharmacological treatment [160].

ECT is considered a first-line treatment in patients with delirious mania and with serious mania, which is associated with potentially lethal physical exhaustion [161,162]. The literature researching the use of ECT in mania is very limited; however, response rates higher than 80% have been reported in acute mania and patients resistant to pharmacological treatment [163,164,165,166]. Due to the efficacy of ECT in the treatment of manic and depressive episodes, its recent use has extended to bipolar disorder mixed states. This condition has been proven to be extremely difficult to treat, with reports of many patients not responding to pharmacological treatment based on the antipsychotics and mood stabilizers used [167,168]. Retrospective studies have shown a response in patients with mixed states treated with unilateral [169] and bilateral [170] ECT. Different naturalistic studies have reported that ECT is an effective treatment for this condition, with response rates higher than 50% [171,172,173]. However, there is a problem in the definition and diagnosis of this state in psychiatry, which makes the available evidence very limited as it is a condition that is commonly underdiagnosed. As a consequence, numerous patients with this condition are included in samples of patients with schizophrenia or mania treated with ECT. Finally, the use of ECT-c and ECT-m in patients with bipolar disorder is limited to very few studies. Despite this, different reports have shown a decrease in hospital admission, hospitalization length, and morbidity in patients with bipolar disorder [17,18,174].

#### 3.3.4. Other Disorders

Due to the positive results seen in refractory bipolar disorder, depression, and schizophrenia, ECT use has recently extended to other neuropsychiatric pathologies. These include PD psychosis, OCD, and PTSD, among others. In the first place, despite that ECT shows efficacy in the treatment of PD motor symptoms [175,176], the amount of substantial clinical evidence is scarce. This is likely due to the inability to predict the therapeutic benefit duration or the established stigma of this intervention [176]. However, its use in PD psychiatric complications has been explored in greater detail during the last years [177,178,179,180]. Borisovskaya et al. [181] performed a systematic review in which 43 studies were included, reporting that depression improved in 93.1% of cases without any negative effects on cognition in the majority of them. Likewise, different studies have researched the beneficial effects in PD psychosis [177,178,179,180]. A retrospective study in which ECT use in PD patients was examined revealed the improvement of motor and nonmotor functions of the patients, including the psychiatric sphere [177]. Furthermore, Ueda et al. [9] observed an improvement in the psychotic symptoms of 4 patients with PD psychosis refractory to antipsychotics. Similar results have been found in different studies [178,179,180].

In regard to OCD, current treatment guidelines do not include ECT as a therapeutic alternative [182,183]. Despite the widespread opinion that its use is not efficient in this disorder, different studies have reported its uses in OCD resistant to treatment. Fontenelle et al. [184] performed a systematic review that included 50 studies and 279 patients, reporting a significantly positive response in 60.4% of the cases. Similar results were found in an assay performed by Maletzky et al. [11] in which long-term positive effects in patients with refractory OCD were observed. In the study, the majority of the subjects showed significant improvement in their symptoms, and this improvement remained 1 year after treatment. Likewise, ECT efficacy in OCD has been reported in different case studies in which, due to a different psychiatric disorder, such as depression or refractory psychosis, ECT was used in patients who had OCD as comorbidity [185,186,187,188,189]. No randomized controlled studies have been performed, and the existing studies have design issues and small samples. Therefore, the evidence that backs its use in patients with OCD resistant to treatment is very limited.

Similarly, the clinical effectiveness and tolerability of ECT in AD have been discussed in the literature, with variable evidence of its use in dementia and agitation [190,191,192]. Sutor et al. [190] published a series of cases that included 11 AD patients over 5 years. They reported agitation improvement or remission, with a significant decrease in the number of long-term hospitalizations. Likewise, Isserles et al. [191] performed a retrospective evaluation of 25 patients with dementia and pre-existing psychiatric disorders who had been treated with ECT. A clinically significant response was reported in 72% of the sessions. Recently, preliminary open-label results suggest that ECT acute treatment is safe and effective to reduce agitation in this population [192]. Two clinical assays are currently in progress. These have the goals of improving cognition by increasing BDNF levels [193] and alleviating dementia conduct and psychological symptoms [194].

Finally, different studies have proposed ECT as an alternative for MNS when pharmacological therapy is not effective [10,195]. In 1987, Hermesh et al. [196] described for the first time an MNS case that responded to ECT. More recently, Morcos et al. [197] performed a retrospective study of a series of cases in which they examined the ECT effectiveness for 17 years. They found that bitemporal ECT was well tolerated and efficient in refractory MNS treatment, with a remission rate of 73.3%. It was suggested that ECT should be used early in cases of refractory MNS, especially if the underlying affliction also responds to ECT.

Similarly, multiple reports have suggested that ECT could benefit PTSD patients. In this sense, in two case reports, the use of multiple ECT sessions led to an improvement of symptoms in two women who were 35 and 38 years old, respectively, and suffering from refractory PTSD [198,199]. Similarly, Watts et al. [200,201] reported in retrospective studies that there was a significant improvement in depressive patients with PTSD after unilateral or bilateral ECT. Likewise, Margoob et al. [12] conducted a prospective study in which 20 patients with refractory PTSD were treated with ECT. It was reported that the mean PTSD score decreased by 34.4% after ECT. Antidepressant treatment was maintained for 6 months during which the improvements also remained. Currently, a clinical randomized assay is being performed [202]. Its goal is to demonstrate ECT efficacy in decreasing traumatic memories in PTSD patients.

## 4. Conclusions

Currently, psychiatric disorders, especially severe affective disorders, continue to be one of the most frequently seen entities in the psychiatric clinical setting, highlighting the need for effective therapeutic options. ECT stands out as a treatment capable of conducting an electric current through the brain to produce a stimulus and trigger a generalized convulsive activity with therapeutic effects. To date, substantial evidence supports the efficacy of ECT in the treatment of different neuropsychiatric disorders. However, the mechanisms of action of this treatment are not completely clear. In this sense, it has been reported that ECT has a multimodal effect by intervening in the immune, endocrine, and neurobiological systems. Furthermore, the role of ECT in the induction of brain structure changes by promoting neurogenesis and regular neuronal interconnectivity through the modulation of the activity of multiple neurotransmitters in the CNS stands out. Likewise, the positive effects of ECT on the HPA and HPT axes and the potential effect on the regulation of the inflammatory response through the release of cytokines and the activation of microglia have also been reported. In this context, it is plausible to consider a potential synergism in the promotion of these mechanisms as a versatile and efficient therapy for the management of these pathologies.

On the other hand, at the clinical level, the research epicenter in the last two decades has been its use in refractory major depressive disorder, and it is considered one of the most effective treatments for this disorder. Likewise, its efficacy in other severe affective disorders, such as schizophrenia and bipolar depression resistant to treatment, has been extensively described. However, during the last years, its use has extended to other mental illnesses, such as PD psychosis, MNS, PTSD, and OCD, with different clinical assays currently in progress [194,202,203]. Regardless, more randomized studies with a more rigorous methodological design and a larger scale are needed to establish ECT as a treatment alternative in these neuropsychiatric disorders.

## Figures and Tables

**Table 1:** Key clinical and preclinical evidence summary regarding ECT and its molecular mechanisms.

		Human Studies *	
**Author**	**Mechanism**	**Methodology**	**Results**
Berggren et al. [25]	Brain interconnectivity	A total of 49 patients underwent ECT. A total of 41 patients grading improvement after the initial ECT series were compared with 8, grading no improvement. The patients underwent neuropsychiatric ratings, the measure of clinical response (defined as ≥50% reduction of pretreatment depression score), and the measure of rCBF.	The responder group had an initial 60–82%, and the nonresponder group a 30–64% clinical response throughout the follow-up. The nonresponder group showed more reported depression (*p* = 0.003) and vegetative anxiety (*p* = 0.024), with a generally higher left temporal rCBF (*p* = 0.045).
Joshi et al. [28]	Neurogenesis	Longitudinal changes in hippocampal and amygdala structures were examined in 43 patients with major depression, referred for ECT as part of their standard clinical care. Cross-sectional comparisons with 32 demographically similar controls established diagnosis effects.	Patients showed smaller hippocampal volumes than controls at baseline (*p* < 0.04). Both the hippocampal and the amygdala volumes increased with ECT (*p* < 0.001) and in relation to the symptom improvement (*p* < 0.01). Hippocampal volume at baseline predicted subsequent clinical response (*p* < 0.05). All structural measurements remained stable across time in controls.
Saijo et al. [52]	Dopaminergic system	A total of 7 patients with depression underwent PET scans before and after a series of 6–7 treatments with the bilateral ECT. The [(11)C]FLB 457 binding parametric images were generated on the basis of a simplified reference tissue model. Voxel-based methods were used to assess the ECT effect on D(2) receptor binding.	There were no significant differences in D(2) receptor binding between patients with depression and controls. Significant changes in D(2) receptor binding, a mean of 25.2% reduction, were found in the right rostral anterior cingulate following ECT (*p* < 0.001).
Burgese et al. [84]	Endocrine effects	Blood cortisol levels were measured before the beginning of treatment with ECT, at the seventh session, at the last session, and at treatment completion. Depression symptoms were assessed using the BDI.	Cortisol levels remained stable between the seventh and the last sessions of ECT; values ranged at 0.686 ± 9.6330 g/dL for women, and there was a mean decrease of 5.825 ± 6.0780 g/dL (*p* = 0.024). After the seventh and the last ECT sessions, patients with depression and individuals in the control group had similar cortisol levels, whereas the BDI scores remained different.
		Animal Studies *	
**Author**	**Mechanism**	**Methodology**	**Results**
Roman et al. [107]	Immunological effects	Wistar rats received single or chronic treatment with ECS, once a day for 10 consecutive days, or sham ECS was administered likewise. The rats were killed 24 h after the last treatment, and peritoneal macrophages were cultured in vitro for a subsequent metabolic activity determination.	We found statistically significant changes in the biological properties of macrophages. Rats receiving chronic 10-fold ECS showed an increase in the macrophages’ metabolic activity, increased arginase activity, and a marked but statistically insignificant decrease in nitric oxide synthesis compared with the respective controls.

* Search strategy: An exhaustive bibliographical search was performed using the terms “electroconvulsive therapy”, “neurobiological effects of electroconvulsive therapy”, “electroconvulsive therapy and immune system”, “electroconvulsive therapy and the endocrine system”, “molecular mechanisms in electroconvulsive therapy”, and “electroconvulsive therapy and psychiatric disorders”. The search was later filtered using the terms “humans” and “animals” as well as “clinical” and “preclinical”. For the selection of the studies, those that were published within the past 35 years were included. Abbreviations: ECT: electroconvulsive therapy; rCBF: regional cerebral blood flow; BDI: Beck Depression Inventory; ECS: electroconvulsive shock.

**Table 2:** Key clinical evidence summary regarding ECT and psychiatric disorders.

Author *	Psychiatric Disorder	Methodology	Results
Diermen et al. [16]	Depression	Meta-analysis with 34 randomized controlled clinical trials evaluating the effects of ECT in patients with major depression.	The presence of psychotic features is a predictor of ECT remission (OR = 1.47, *p* = 0.001) and response (OR = 1.69, *p* < 0.001), as is older age (SMD = 0.26 for remission and 0.35 for response *p* < 0.001). The severity of depression predicts response (SMD = 0.19, *p* = 0.001) but not remission.
Elias et al. [135]	Depression	Meta-analysis with 5 randomized controlled clinical trials that assessed the efficacy of continuation ECT and maintenance ECT in preventing relapse and recurrence of depression.	Continuation ECT and maintenance ECT with pharmacotherapy were associated with significantly fewer relapses and recurrences than pharmacotherapy.
Ahmed et al. [19]	Schizophrenia	Meta-analysis with 9 randomized controlled clinical trials evaluating the effects of TEC in patients with resistant schizophrenia.	The ECT augmentation technique was found to be effective in the reduction of psychometric scale scores, and the resulting improvement was significant.
Bahji et al. [137]	Bipolar depression	Meta-analysis with 19 randomized controlled clinical trials evaluating the effects of TEC in patients with bipolar disorder in a resistant depressive episode.	The pooled response and remission rates with TEC in bipolar depression were 77.1% (n = 437/567) and 52.3% (n = 275/377), respectively. Response rates to TEC were statistically higher in bipolar depression than in unipolar depression (OR = 0.73, 95% CI: 0.56–0.95, *p* = 0.02).
Ueda et al. [9]	PDP	Retrospective study evaluating the influence of acute ECT on PDP.	The psychosis scores after ECT improved significantly compared with those before ECT.
Maletzky et al. [11]	OCD	Systematic review of 50 articles reporting the efficacy of the acute treatment of ECT for OCD.	A positive response was reported in 60.4% of the 265 cases that were studied.
Margoob et al. [12]	PTSD	An open, prospective study evaluating the influence of ECT in patients with severe, chronic, extensive antidepressant-refractory PTSD.	Scores evaluating PTSD significantly decreased by a mean of 34.4%.

* Search strategy: An exhaustive bibliographical search was performed using the terms “electroconvulsive therapy”, “neurobiological effects of electroconvulsive therapy”, “electroconvulsive therapy and immune system”, “electroconvulsive therapy and the endocrine system”, “molecular mechanisms in electroconvulsive therapy”, and “electroconvulsive therapy and psychiatric disorders”. The search was later filtered using the terms “humans” and “animals” as well as “clinical” and “preclinical”. For the selection of the studies, those that were published within the past 35 years were included. Abbreviations: ECT: electroconvulsive therapy; PDP: Parkinson’s disease psychosis; OCD: obsessive–compulsive disorder; PTSD: post-traumatic stress disorder.

## Data Availability

Not applicable.
